# The Effects of an Ocular Direct Electrical Stimulation on Pattern-Reversal Electroretinogram

**DOI:** 10.3389/fnins.2020.00588

**Published:** 2020-06-10

**Authors:** Maren-Christina Blum, Alexander Hunold, Benjamin Solf, Sascha Klee

**Affiliations:** Institute of Biomedical Engineering and Informatics, Technische Universität Ilmenau, Ilmenau, Germany

**Keywords:** ocular electrical stimulation, direct current stimulation, non-invasive brain stimulation, electroretinogram, pattern-reversal ERG

## Abstract

Studies on transcranial current stimulation have shown that a direct current stimulation of the occipital cortex can influence the amplitude size of a visual evoked potential (VEP). The current direction (cathodal or anodal) determines whether the VEP amplitudes increase or decrease. The aim of this study was to design a new experimental setup that will enable a simultaneous ocular direct current stimulation and electroretinogram (ERG) recording which will broaden our understanding of current stimulation effects on the visual system. Furthermore, we examined whether a direct current stimulation on the eye has a similar effect on an ERG as on a VEP. The pattern-reversal ERG was measured with sintered Ag/AgCl skin-electrodes, positioned on the lower eyelid (active), the earlobe (reference), and the forehead (ground). Direct current was applied through a ring rubber electrode placed around the eye and a 5 cm × 5 cm rubber electrode placed at the ipsilateral temple with a current strength of 500 μA and a duration time of 5 min. Fifty-seven healthy volunteers were divided into three groups depending on the current direction (cathodal, anodal, and sham stimulation, *n* = 19 each). One ERG measurement (ERG 1) was performed before and another (ERG 2) during the direct current stimulation. The difference between ERG 1 and ERG 2 measurements for the characteristic P50, N95 and N95′ (N95 minimum measured from zero line) amplitudes were evaluated by both confidence interval analysis and *t*-test for related samples (α = 0.05, after Bonferroni correction *p*^∗^ = 0.0055). The P50 amplitude was significantly decreased for ERG 2 measurement in the cathodal and anodal stimulation group (cathodal *p* = 0.001, anodal *p* = 0.000). No significant changes could be found in the N95 and N95′ amplitudes as well as in the sham-stimulation group. Additionally, the latencies did not undergo any significant changes. In conclusion, the newly designed experimental setup enables simultaneous current stimulation and ERG recording. The current influenced P50 amplitude although not the N95 and N95′ amplitudes. Furthermore, the amplitude size decreased for both current directions and did not lead to contrary effects as expected.

## Introduction

In recent years, ocular electrical stimulation (ES) with an alternating current of 1–2 mA has gained attention. Notably, positive effects such as an increase in the levels of the various growth factors that influence the development, function and stability of retinal nerve cells have been detected in animal studies (Morimoto et al., 2005; Sato et al., 2008a, b; Ni et al., 2009; Bola et al., 2014; Sergeeva et al., 2015). These results suggest that an ocular ES can cause stabilization and regeneration along the visual pathway (Sehic et al., 2016). Previously, human studies mainly investigated the effect of ocular ES on neurodegenerative eye diseases like glaucoma, retinitis pigmentosa, Stargardt disease, age-related macular degeneration, and retinal artery occlusions or ischemic optic atrophy (Gall et al., 2011; Fedorov et al., 2011; Sabel et al., 2011; Schatz et al., 2011; Naycheva et al., 2013; Röck et al., 2013, 2014; Bola et al., 2014). The stimulation parameters such as frequency, current strength, and configuration of the stimulation electrodes vary widely in the literature. The stimulation induced processes within the retina and visual pathways are not fully understood yet (Freitag et al., 2019, 2020).

It is proven by transcranial ES that a direct current applied to the head causes polarization of neuronal tissue thereby leading to modulation of neuronal activity (Nitsche et al., 2008). A number of studies have found that direct current stimulation of the visual cortex has an effect on the characteristic amplitudes of the visual evoked potential (VEP; Antal et al., 2004; Accornero et al., 2007; Ding et al., 2016; Wunder et al., 2018). Antal et al. (2004) were able to achieve a significant reduction of the N75 amplitude after a cathodal stimulation of the visual cortex (Oz–Cz electrode position, related to the 10–20 system). However, anodal stimulation resulted in an increase in the amplitude (statistically not significant) (Antal et al., 2004). Wunder et al. (2018) found a similar result for cathodal stimulation in there study whereby the N75 amplitude was significantly decreased 1 min after the direct current stimulation. They also found a significant reduction of the P100 amplitude during the stimulation (Wunder et al., 2018). A study by Ding et al. (2016) showed that anodal stimulation leads to an increase in the P100 amplitude following termination of the current stimulation. However, only a trend for an amplitude reduction for the cathodal direction was found (Ding et al., 2016). Similarly, Accornero et al. (2007) stimulated the visual cortex and simultaneously measured the VEP with the counter electrode for the stimulation placed at the neck instead of the scalp. Here, an inverse effect was found in comparison to the studies above. The P100 amplitude increased during cathodal stimulation and decreased during anodal stimulation (Accornero et al., 2007). Further, studies by Strigaro et al. (2015) and Viganò et al. (2013) indicated no effects on the amplitudes (Viganò et al., 2013; Strigaro et al., 2015). The contradictory results can likely be attributed to the different stimulation and measurement setups, which limit the comparability of the studies.

To advance our understanding of ES effects on the visual system, we herein present a study focusing on the electroretinogram (ERG). Electroretinogram allows us to investigate the effects of ES on initial processes of the visual system. A specifically adapted setup was designed to fulfill the technical requirements of simultaneous current stimulation and ERG recording that included the following: the amplifier system should be galvanically separated from the rest of the experimental setup, it should operate in battery mode, and have a high dynamic range and a high-resolution analog-to-digital converter (minimum: 24 bit) (Fehér and Morishima, 2016). Further, sintered Ag/AgCl electrodes should be used as recording electrodes because of their long-term stability, low-frequency noise, and their stability against polarization effects (Woods et al., 2015). Additionally, the current stimulator should be battery powered and the impedance of the current electrodes must be kept as low as possible because a high load resistance will result in a high additional noise level, which will interact with the ERG (García-Cossio et al., 2017). Furthermore, no bridges between the ERG and the current electrodes are allowed.

Similar to the effect of direct current stimulation on the VEP, we hypothesized a change in the characteristic ERG amplitudes with opposite effects (increase or decrease) for different current polarities.

## Materials and Methods

### Participants

Sixty-six healthy subjects (mean age: 26.9 ± 6.6 years, 28 females, 38 males) participated in the study. All volunteers were asked about their state of health and provided written informed consent. Exclusion criteria included the following: neurological, eye, skin, or heart diseases; metal implants in the head area; allergies or hypersensitivities of the skin, and pregnancy. The volunteers were randomly divided into three independent groups (cathodal: *n* = 26, anodal: *n* = 20, and sham: *n* = 20). Poor quality ERG measurements (no typical amplitudes could be measured) due to bad electrode contact in four subjects and a technical problem in five subjects led to the exclusion of 9 subjects from the analysis. On completion of the study, each group (cathodal, anodal, and sham stimulation) contained evaluable data sets for 19 subjects (mean age: 26.8 ± 6.7 years, 24 females, 33 males). The study was prepared in accordance with the Declaration of Helsiniki and was approved by the Ethics commission at the medical faculty of the Friedrich-Schiller-University Jena, Germany.

### Electroretinogram Recording

An ERG recording monitored the activation of retinal ganglion cells provoked by a pattern-reversal stimulus. The pattern-reversal checkerboard (visual field size = 16.2°, individual square size = 1°, reversals per second = 4, Michelson contrast = 99%, mean luminance = 186 cd/m^2^) was presented binocularly on a 52 inches LCD display (LE52F96BD, Samsung Group, Seoul, South Korea) with a resolution of 1920 pixels × 1080 pixels. The subjects were seated 45 cm in front of the screen and were light adapted. Low visual acuity (±2 diopter) was compensated with suitable corrective lenses to obtain a sharp image of the visual stimulus. To avoid movement artefacts, the head was placed in a height-adjustable head and chin rest. In total, 900 sweeps per measurement were recorded, which corresponds to a measurement time of 3 min 45 s. While recording, the subject had to look at a fixed red point in the center of the changing checkerboard.

A *TheraPrax* amplifier system (neuroCare Group GmbH, Munich, Germany) recorded the pattern-ERG with a dynamic range of ±140 mV, a 24- bit analog-to-digital converter, an input impedance of ≥10 GΩ, and a sampling rate of 1024 sps. Sintered Ag/AgCl ring electrodes were used to detect the ERG. The active electrode was placed on the lower eyelid, while the reference electrode was attached to the ipsilateral earlobe and the ground electrode was placed on the forehead of the volunteer. To ensure good signal quality the skin at the electrode positions was prepared with *NuPrep* contact-gel (Weaver and Company, Aurora, CO, United States) and the electrodes were coated with *Ten-20* conductive EEG paste (Weaver and Company, Aurora, CO, United States). Further, the electrodes were fixed with roller pavement.

### Ocular Direct Current Simulation

**FIGURE 1:**
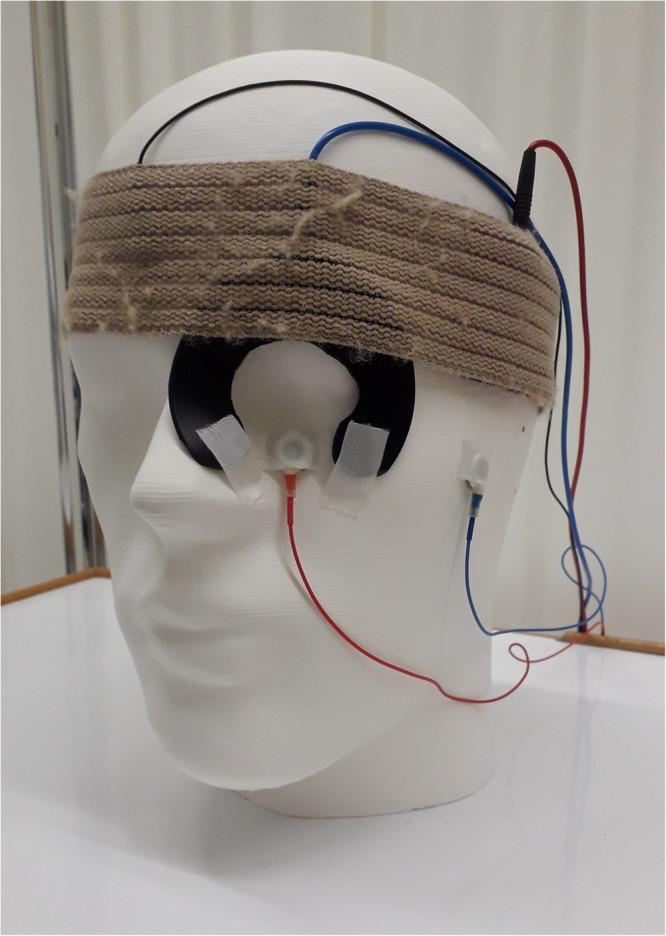
Electrodes and their positions for a simultaneous electroretinogram (ERG) measurement and an ocular direct current stimulation. For current stimulation, a ring rubber electrode was cut to size and positioned around the eye to prevent the establishment of a bridge to the active skin ERG electrode at the lower eyelid. A standard square rubber electrode in combination with a saline-soaked sponge was used as a counter electrode and was positioned at the ipsilateral tempus. The ERG reference electrode was placed at the earlobe and the ground electrode at the forehead.

Direct current was applied using the DC-Stimulator MC (neuroCare Group GmbH, Munich, Germany) that was powered by battery extension. A ring rubber electrode with an outer diameter of 75 mm, an inner diameter of 30 mm, and a thickness of 2 mm was used in combination with *Ten20* conductive gel to feed current into the eye. The rubber electrode had a cutout in the area of the lower eyelid (shown in Figure 1) to allow placement of the ERG electrode, which was fixed with roller pavement. Before applying the counter electrode, the subject’s hair was lightly moistened with saline solution to achieve the necessary low impedance. The counter rubber electrode (5 cm × 5 cm) was placed in a saline-soaked (10 ml) sponge and positioned at the ipsilateral tempus with a fixation strap. Either anodal, cathodal, or sham stimulation was performed during the study, wherein polarity refers to the stimulation electrode around the eye.

Before each current stimulation, an impedance test was carried out according to the protocol provided along with the current stimulator (sinus, 200 μA peak-to-peak, 20 Hz). To start a stimulation, the impedance between stimulation electrodes had to be less than or equal to 8 kΩ (was not achieved in 2 volunteers). The stimulation current was increased from 0 to 500 μA in 5 s at the start of the stimulation to prevent transient sensations. The current intensity (500 μA) was chosen such that it is slightly above the mean flicker response of healthy subjects (Freitag et al., 2019). The direct current was kept constant for 5 min and was subsequently ramped down in 5 s.

When performing sham stimulation, the impedance test was performed to give the subject a feeling that current was really applied. However, through the duration of the 5 min current stimulation no active current source was attached to the electrodes. The volunteer was only informed that the stimulation had started. All volunteers were informed that the current perceptions are individual-based and that a tingling sensation can occur underneath the electrodes.

### Experimental Procedure

First, the skin underneath the Ag/AgCl electrodes was cleaned with alcohol. Next, the ERG recording electrodes, the ring stimulation electrode, and the counter stimulation electrode were fixed (electrode positions are shown in Figure 1). The wiring of the electrodes to the current stimulator determined the cathode and the anode positions. Two ERG recordings were performed: one before (ERG 1) and one during (ERG 2) the current stimulation. ERG 1 represented the baseline measurement. Following the baseline measurement, the impedance test was carried out and subsequently the current or sham stimulation was executed. One minute after the start of the stimulation the second ERG measurement was performed. All the experiments were conducted by the same individual.

### Signal Processing

Signal Processing was performed with MATLAB, version 2019 (The Mathworks, Inc., Natick, MA, United States). The ERG raw signal was filtered forward and backward to avoid phase shifting with an infinite impulse response (IIR) high pass (Butterworth, filter order: 3, half power frequency: 0.75 Hz) and low pass (Butterworth, filter order: 10, half power frequency: 35 Hz) filter. The sweeps that contained amplitudes greater than 30 μV after the filtering process were evaluated as artefact afflicted. For the remaining sweeps, the Pearson correlation was calculated to a template, which consisted of 60 averaged ERG signals (from preliminary studies with this measurement setup). Altogether 600 sweeps with the highest correlation coefficient were averaged for each subject. The averaged signal was centered at the time point zero to the amplitude zero. The P50 amplitude was defined as the maximum of the averaged ERG between 25 and 75 ms after the pattern change with a height measured from the zero line. N95 amplitude was determined as the minimum between 70 and 120 ms after the pattern change and measured from the P50 peak. Moreover, the N95′ amplitude as measured from zero line to the minimum deflection between 70 and 120 ms was analyzed.

### Statistical Analysis

Statistical analysis was performed using IBM SPSS Statistics, version 25 (IBM Corp., Armonk, NY, United States). To perform statistical analysis the amplitude differences between the components from ERG 1 and ERG 2 measurements were determined. The significance level was set to α = 0.05. The normal distribution hypothesis was not rejected by the Shapiro–Wilk test (Table 1). Therefore, *t*-test for related samples and the *t*-test based confidence interval analysis were used. Nine statistical tests were performed to analyze the effects on the amplitude measures (P50, N95, and N95′) in the three stimulation conditions (cathodal, anodal, and sham). Concerning the connected tests, a Bonferroni correction resulted in an adjusted significance value of *p*^∗^ = 0.00555. The effect strength was determined using the Cohens *d* value (Cohen, 1988). The grand mean signal was calculated for ERG 1 and ERG 2 measurements in every group for graphical evaluation. In addition, violin plots were created, which showed the distribution of the different values of the subjects within a group.

**TABLE 1:** Shapiro–Wilk test results (*p*-values) for verification of normal distribution of the measured amplitudes.

**Shapiro–Wilk test**	**P50 cathodal**	**N95′ cathodal**	**N95 cathodal**	**P50 anodal**	**N95′ anodal**	**N95 anodal**	**P50 sham**	**N95′ sham**	**N95 sham**
*p*-value	0.847	0.753	0.233	0.969	0.229	0.488	0.747	0.228	0.869

*The significant level was set to α = 0.05.*

## Results

Our new designed experimental setup comprising a bio-signal amplifier with a 24- bit resolution on a range of ±140 mV, a battery powered constant current source and specially adapted electrode shapes, enabled us to measure pattern-reversal ERG simultaneously with ocular direct current stimulation. The mean values and standard deviation for the ERG component amplitudes and latencies are listed in Table 2. Regarding latencies, no significant differences could be observed. The calculated *p*-values and confidence intervals are summarized in Table 3.

**TABLE 2:** Mean values and standard deviation of the ERG component amplitudes and latencies.

		**P50 amplitude in μV**	**P50 latency in ms**	**N95′ amplitude in μV**	**N95′ latency in ms**	**N95 amplitude in μV**	**implicit time in ms**
Cathodal	ERG 1	1.836 ± 0.708	33.563 ± 3.778	−2.459 ± 1.004	82.391 ± 7.606	4.295 ± 1.137	48.828 ± 6.313
	ERG 2	1.529 ± 0.606	33.512 ± 4.132	−2.530 ± 0.980	84.344 ± 10.08	4.059 ± 1.175	50.833 ± 10.793
	Difference	−0.307 ± 0.337	−0.051 ± 2.912	0.065 ± 0.428	1.953 ± 10.532	−0.236 ± 0.42	2.005 ± 11.496
Anodal	ERG 1	1.803 ± 0.694	33.563 ± 3.459	−2.686 ± 0.721	85.578 ± 6.738	4.489 ± 1.184	52.015 ± 6.645
	ERG 2	1.298 ± 0.547	31.918 ± 4.436	−2.952 ± 0.884	84.036 ± 6.931	4.250 ± 1.051	52.118 ± 6.186
	Difference	−0.504 ± 0.442	−1.645 ± 3.089	0.132 ± 0.729	−1.542 ± 7.665	−0.239 ± 0.618	0.103 ± 6.809
Sham	ERG 1	1.875 ± 0.820	33.663 ± 3.401	−2.937 ± 0.644	83.008 ± 6.355	4.813 ± 0.888	49.345 ± 5.636
	ERG 2	1.727 ± 0.598	33.49 ± 3.771	−3.052 ± 0.742	84.501 ± 6.641	4.779 ± 0.776	51.011 ± 5.768
	Difference	−0.142 ± 0.372	0.051 ± 2.697	0.057 ± 0.337	1.131 ± 2.675	−0.086 ± 0.407	1.079 ± 3.214

**TABLE 3:** *P*-values and confidence intervals for the difference between the two ERG measurements.

		***p*-value**	**Confidence interval lower limit (μV)**	**Confidence interval upper limit (μV)**
Cathodal	P50	0.001*	−0.55*	−0.063*
	N95′	0.483	−0.24	0.374
	N95	0.024	−0.54	0.067
Anodal	P50	0.000*	−0.823*	−0.185*
	N95′	0.06	−0.396	0.659
	N95	0.109	−0.685	0.208
Sham	P50	0.113	−0.441	0.127
	N95′	0.473	−0.187	0.3
	N95	0.371	−0.38	0.209

*The significance level was set to α = 0.05. After Bonferroni correction the adjusted significance value was ^∗^*p* = 0.00555. The confidence interval of the difference between the ERG measurements is significant if the interval excludes zero.*

### Results of Cathodal Stimulation

**FIGURE 2:**
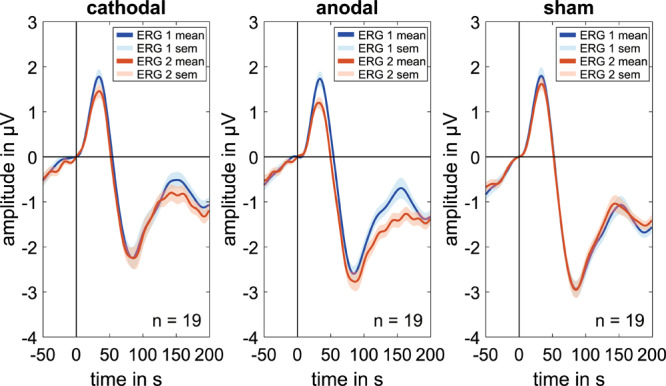
Grand mean signals for every stimulation group (cathodal, anodal, and sham; *n* = 19 for each curve) for the baseline measurements (ERG 1, blue curve), which is done before the current stimulation and the second ERG-measurements (ERG 2, orange curve), which is done during the ocular direct current stimulation. Concerning the P50 amplitude, every group shows a visible decrease in the ERG 2 measurements whereas the N95 amplitude shows an increasing effect for the anodal stimulation. Due to latency time differences between the subjects, smaller amplitudes could have occurred in the grand mean diagrams. Therefore, the grand mean signals show only a trend for the amplitude changes.

Cathodal stimulation resulted in a significant decrease in the P50 amplitudes in ERG 2 when compared with ERG 1 (Figure 2) while there was a marginal increase in the N95′ amplitudes. This resulted in a decrease in the N95 amplitude.

**FIGURE 3:**
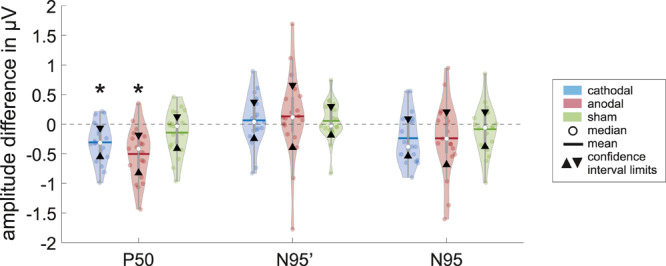
Violins show the data distribution of the measured differences inclusive the box-and-whisker plot (25 and 75% quartiles represented by the gray boxes and whiskers by the gray lines) of the P50, N95′ (N95 minimum measured from zero line), and N95 amplitudes for the different groups (cathodal, anodal, or sham-stimulation; *n* = 19 for each plot) with statistical measures. The significant level of α = 0.05 is Bonferroni-corrected such that the new significant *p*-value is *p*^∗^ *<* 0.0055. The confidence interval (area within triangles) for the difference in the P50 amplitude between ERG 1 and ERG 2 measurement do not include zero, which indicates that there is a significant effect on the ERG for a direct current stimulation (marked with ^∗^). Also, the *t*-test for related samples was significant (cathodal: *p* = 0.001^∗^, anodal: *p* = 0.000^∗^).

The mean of P50 amplitude decreased by −0.307 ± 0.337 μV in the ERG 2 measurement when compared with ERG 1. The confidence interval ([lower; upper limit] = [−0.55 μV; −0.063 μV]^∗^) of this difference indicated a significance when excluding the zero level (Figure 3). Further, *t*-test for related samples showed a significant decrease (*p* = 0.001^∗^). The effect strength was *d* = 0.91.

The N95′ amplitude increased by 0.065 ± 0.428 μV on average. The confidence interval including zero ([−0.244 μV; 0.374 μV]) indicated no significant difference between ERG 1 and ERG 2 measurements. Consistently, the *t*-test did not show a significant difference between the two measurements (*p* = 0.483).

The mean difference in the N95 amplitude between the two measurements was −0.236 ± 0.42 μV. The confidence interval included zero ([−0.54 μV; 0.067 μV]) thereby indicating no statistically significant effect. Further, the *t*-test showed no significant difference (*p* = 0.024).

### Results of Anodal Stimulation

Regarding anodal stimulation, the P50 amplitude showed a distinct decrease in the grand mean (Figure 2). The N95′ amplitude increased marginally while the N95 amplitude decreased because of the larger effect in the P50 amplitude. These outcomes were comparable to the effects resulting from cathodal stimulation. Confidence interval analysis (P50: [−0.823 μV; −0.185 μV]^∗^, N95′: [−0.395 μV; 0.659 μV], and N95: [−0,685 μV; 0.208 μV]) as well as *t*-test (P50: *p* = 0.000^∗^, N95′: *p* = 0.06, and N95: *p* = 0.109) showed a significant reduction with an effect strength of *d* = 1.14 for the P50 amplitude while no significant effects could be found for the N95′ and N95 amplitudes. The decrease in P50 amplitude was −0.504 ± 0.442 μV on average and was thus larger than the effect of the cathodal stimulation. The N95′ and N95 amplitudes showed a mean difference of 0.132 ± 0.729 μV and −0.239 ± 0.618 μV, respectively.

### Results of Sham Stimulation

ERG 2 measurements of the sham group showed a small decrease in the grand mean of P50 and N95 amplitudes and a small increase in the N95′ amplitude when compared with ERG 1. However, the changes were not as pronounced as in the other two groups. Statistical analysis indicated no significant differences between the ERG measurements (confidence interval: P50: [−0.411 μV; 0.127 μV], N95′: [−0.187 μV; 0.3 μV], and N95: [−0.38 μV; 0.209 μV]; *t*-test: P50: *p* = 0.113, N95′: *p* = 0.473, and N95: *p* = 0.371). On average the P50 and N95 amplitudes decreased by −0.142 ± 0.372 μV and −0.086 ± 0.407 μV, respectively. The N95′ amplitude increased by 0.057 ± 0.337 μV.

## Discussion

For the first time, we were able to perform a simultaneous pattern-reversal ERG measurement and an ocular direct current stimulation with a newly designed experimental setup that made it possible to examine stimulation effects on the ERG amplitudes and latencies during current stimulation. We first measured baseline ERG and subsequently stimulated one eye with a cathodal, anodal, or sham stimulation (three independent groups, *n* = 19 each) while simultaneously performing an ERG measurement and compared the result with the first measurement. We expected amplitude changes with polarity-dependent opposing effects and no influence of sham stimulation on the ERG. As expected, no significant results were found for sham stimulation. Concerning both cathodal and anodal stimulation, the P50 amplitude decreased significantly during stimulation. The N95′ and N95 amplitudes showed no significant stimulation effects.

Based on earlier studies that investigated cathodal transcranial ES effects on VEPs, amplitude reductions in recorded wave forms were expected, as described for the N75 (Antal et al., 2004) and P100 (Ding et al., 2016; Wunder et al., 2018) components of the VEP. In our pattern ERG study, we likewise found a significant reduction in the P50 amplitude during cathodal ocular direct current stimulation. Thus, the results of our present study are congruent with previous publications.

We expected opposing amplitude effects when inverting the stimulation polarity. This has been shown previously in studies investigating transcranial ES effects on VEPs (Antal et al., 2004; Accornero et al., 2007; Ding et al., 2016; Wunder et al., 2018). However, the P50 amplitude decreased significantly for cathodal as well as for anodal stimulation.

Two theories could serve as potential explanations of this polarity independent effect.

One theory is based on the origin of pattern ERG amplitudes. The characteristic pattern-reversal ERG amplitudes have different origins (Bach and Hoffmann, 2006). Humans with full optic nerve atrophy have no N95 amplitude but a reduced P50 amplitude (Bach et al., 1992; Holder, 2001). This suggests that the P50 amplitude results from excitatory/inhibitory postsynaptic potential in the ganglion cells and from other pre-ganglion cells. The N95 amplitude however is the result of output activity of the ganglion cells (Bach and Hoffmann, 2006). We perhaps did not apply direct current stimulation only on the ganglion cells but also on other retina layers, which may have an additional influence on the P50 activity. The combination of an ocular ES and a flash ERG, which reflect the activity of neurons located more deeply in the retina, could lead to different results. The second theory depends on the processing of the checkerboard signal. When the pattern checkerboard is on, a bright square indicates a local ON response while the neighboring black square indicates an OFF response. When the ON and OFF responses are alike with different deflections, the temporal addition of the two responses annihilate each other and ERG would not be measurable. But the ON response is weighted slightly more resulting in a measurable ERG (Bach and Hoffmann, 2006). Direct current stimulation could have a different influence on the ON and OFF responses for different polarities such that their settlement always results in a reduction of the P50 amplitude. Potentially, the cathodal stimulation only decreased the ON response and the anodal polarity only increased the OFF response. Although the current had contrasting influences, the amplitudes of the ERG decreased for both polarities. A flash stimulation could evoke different results because the generated ERG is a pure sum signal where all activated cells generate the same response such that no extinguishing effects occur.

The effect of current depends primarily on the current direction as demonstrated by the contradictory results of Accornero et al. (2007) who placed the counter electrode at the neck instead of at the scalp in contrast to the electrode placement in other studies (Antal et al., 2004; Accornero et al., 2007; Ding et al., 2016; Wunder et al., 2018). In our measurement setup, the whole eye was not stimulated consistently because of both the cutout in the ring electrode for ERG recording at the lower eyelid and the position of the counter current electrode, which was located at the ipsilateral temple to prevent the pathway of current through the entire brain and the visual cortex. However, it is known from previous studies that a counter electrode position at the back of the head would produce a more homogeneous current flow through the entire eye and the optic nerve (Hunold et al., 2015). Hence, we supposedly stimulated the temporal eye further with our electrode positions. However, the axons of the ganglion cells, which converge at the optic nerve, are located in a more nasal position. Accordingly, other electrode positions that lead to other current pathways could influence the results. Additionally, the stimulation parameters such as current strength and duration have considerable influence. To maintain a low current load, we stimulated the subjects with 500 μA current for 5 min. Higher current intensities and longer stimulation times can have a different effect on the ERG. The impact of stimulation parameters was reviewed in applications to influence cognitive processes, such as schizophrenia (Kostova et al., 2020) and attention (Reteig et al., 2017). It was noticed that all studies differed considerably in the experimental design and stimulation protocols and they could not identify stimulation parameter which clearly mediated differences in cognitive outcomes. A thorough investigation of the stimulation parameter space is desired to identify parameter combinations that allow outcome discriminations in several applications of ES.

Potential after-effects of the current stimulation were not investigated. Further, the effects of current stimulation on the amplitudes may still occur after termination of the stimulation as reported in previous studies (Antal et al., 2004; Accornero et al., 2007; Ding et al., 2016; Wunder et al., 2018). For instance, Wunder et al. (2018) found a decrease in the P100 VEP amplitude during stimulation. Nevertheless, this effect diminished subsequently. However, there was a reduction in the N75 amplitude after the current stimulation. It is known that a direct current stimulation modulates neurotransmitter (Kostova et al., 2020), e.g., glutamate and gamma-Aminobutyric acid, and that these modulations are associated with after-effects (Das et al., 2016; Kuo et al., 2016). Also, the retinal cells communicate via neurotransmitter changes and modulations of them could influence the ERG. The investigation of after-effects and neurotransmitter alternations within the retina during and after an ocular ES would be important for future understanding.

During the measurements performed in the present study, adaptation processes could potentially lead to amplitude reductions. In this case, all measurements would be equally affected. The data presented here showed no significant amplitude reduction between ERG 1 to the ERG 2 measurement.

In summary, for the first time we were able to simultaneously measure an ERG during the application of direct current stimulation on the eye. During stimulation, the P50 amplitude decreased significantly for both cathodal and anodal direct current stimulation whereas the N95 amplitude was not influenced. This leads to the conclusion that an ocular ES has a measurable effect on the retinal cells.

## Data Availability Statement

The datasets generated for this study are available on request to the corresponding author.

## Ethics Statement

The studies involving human participants were reviewed and approved by Ethikkommission der Friedrich-Schiller-Universität Jena an der Medizinischen Fakultät. The patients/participants provided their written informed consent to participate in this study.

## Author Contributions

M-CB: conceptualization, methodology, data acquisition/curation, data processing/analysis, manuscript drafting, and manuscript revision. AH and BS: conceptualization, methodology, and manuscript revision. SK: project administration/supervision, conceptualization, methodology, and manuscript revision.

## Conflict of Interest

The authors declare that the research was conducted in the absence of any commercial or financial relationships that could be construed as a potential conflict of interest.
